# Prognostic implications of impaired three-dimensional left atrial function and stiffness in primary mitral regurgitation

**DOI:** 10.1186/s12947-026-00368-4

**Published:** 2026-02-16

**Authors:** Christian E. Berg-Hansen, Rasmus Bach Sindre, Lisa M. D. Grymyr, Cecilie Linn Aas, Stig Urheim, Judy Hung, Dana Cramariuc

**Affiliations:** 1 Department of Heart Disease, Haukeland University Hospital, Jonas Lies vei 65, Bergen, NO-5021 Norway; 2 Department of Clinical Science, University of Bergen, Bergen, Norway; 3 Division of Cardiology, Cardiac Ultrasound Laboratory, Massachusetts General Hospital, Harvard Medical School, Boston, MA USA

**Keywords:** Mitral regurgitation, 3D echocardiography, Left atrial strain, Left atrial stiffness, Mitral valve

## Abstract

**Aims:**

In patients with mitral regurgitation (MR), cardiac remodeling by two-dimensional (2D) echocardiography is variable and less suitable for individualized risk assessment. We evaluated whether three-dimensional (3D) peak LA reservoir strain (LASr) and stiffness improve risk prediction in primary MR.

**Methods:**

In the prospective 3D Echocardiography and Cardiovascular Prognosis in Mitral Regurgitation (3D-PRIME) study, 110 patients with moderate or greater primary MR underwent 2D/3D echocardiographic assessment of LASr and LA stiffness (i.e. (mitral E-wave/ annular e’ velocity)/ LASr). The primary outcome was a composite of death, heart failure worsening and mitral valve intervention.

**Results:**

During 24 [17–26] months follow-up, the primary outcome occurred in 59 patients. In multivariable Cox analyses, low 3D LASr (HR 2.1, 95% CI 1.1–4.3) and increased 3D LA stiffness (HR 3.5, 95% CI 1.6–7.4) predicted higher risk of adverse events after adjustment for left ventricular (LV) global longitudinal strain (GLS), MR severity, LV end-systolic volume and maximum LA volume (*p* < 0.05). In likelihood ratio tests, 3D LASr or LA stiffness (but not the corresponding 2D indices) increased the predictive value of a model including LV GLS and current indications for intervention in primary MR (χ^2^ -increase from 58 to 63 / 70, *p* < 0.05).

**Conclusion:**

In moderate or greater primary MR, impaired LA function by 3D echocardiography is associated with higher risk of disease progression towards death, worsening heart failure and mitral valve intervention. Our findings lay the groundwork for future multicenter studies to explore the value of routine assessment of 3D LA remodeling in the follow-up of patients with primary MR.

**Graphical abstract:**

3D LA strain and stiffness and clinical outcomes in primary MR

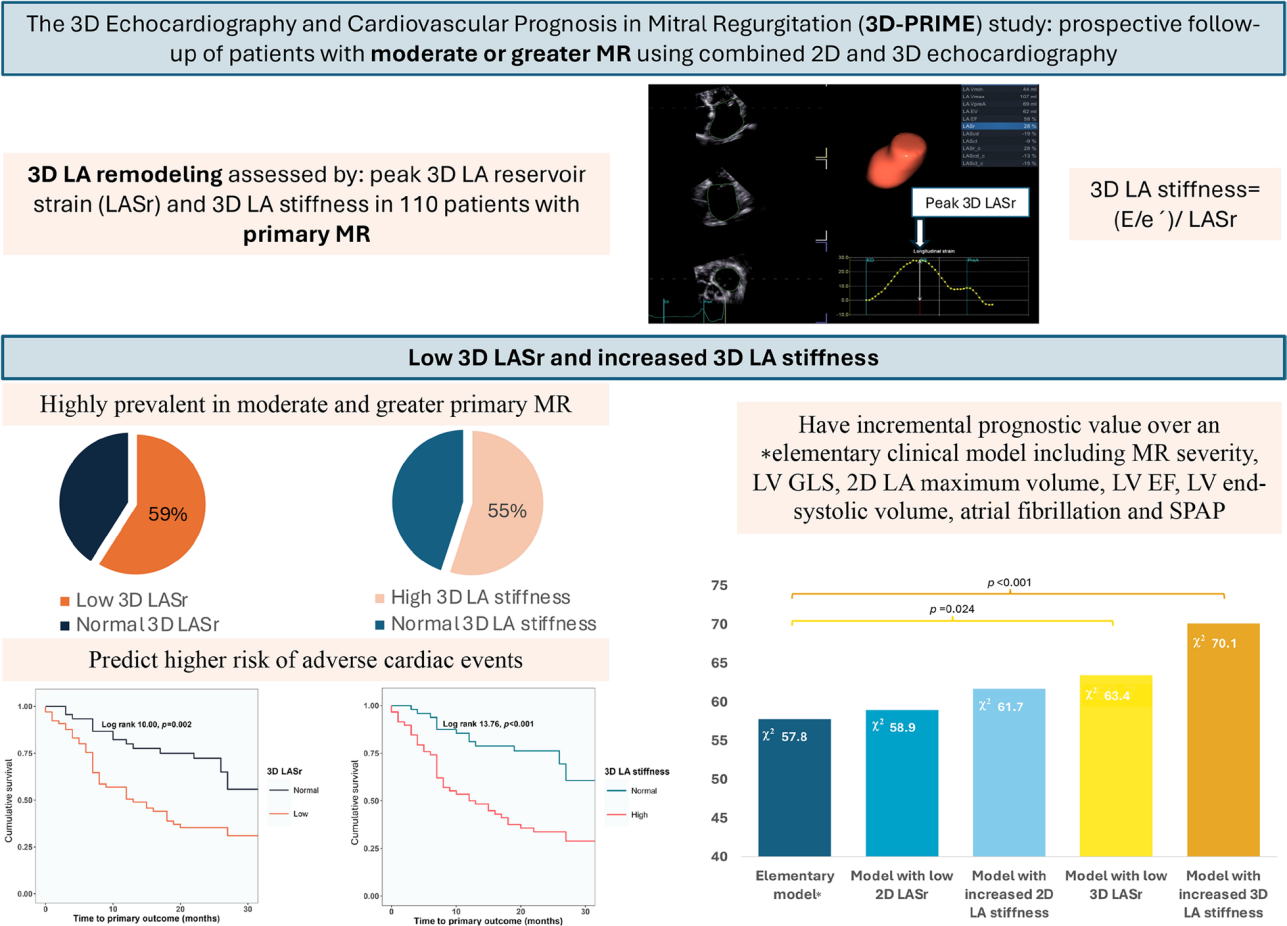

## Introduction

 Mitral regurgitation (MR) leads to chronic volume overload of the left atrium (LA) resulting in progressive structural, mechanical and electrical LA remodeling. An enlarged LA, measured by either its maximal diameter or two-dimensional (2D) volume (LAV), has been shown to adversely impact survival in patients with primary MR, as evidenced by several retrospective and prospective analyses [1–3]. These findings have prompted the inclusion of increased maximum 2D LA size among the indications for intervention in primary MR within the current European guidelines for management of valvular heart disease [4]. However, assessment of LA remodeling using 2D indices often yields inconsistent results in clinical practice, primarily due to geometric assumptions that may not accurately capture the three-dimensional (3D) complexities of the LA in individual patients. This limitation can be addressed through the use of 3D echocardiography, which avoids geometrical simplifications and provides measurements of LA remodeling that correlate more closely with findings from cardiac magnetic resonance imaging in healthy individuals [5].

Prior retrospective studies have found impaired LA reservoir strain (LASr) to be linked to higher mortality rates in both primary and secondary MR [6–8]. Limited histopathological and echocardiographic data suggest that impaired LA reservoir function might be due to accumulation of fibrosis in the LA wall in the setting of chronic volume overload. Increased fibrosis may lead to stiffening of the LA and reduced wall compliance during the reservoir phase [9]. In the 3D Echocardiography and Cardiovascular Prognosis in Mitral Regurgitation (3D-PRIME) study, we demonstrated earlier that one-third of patients with moderate or greater MR had elevated echocardiographic indices of LA stiffness, with women presenting lower LASr and higher stiffness values compared to men despite having comparable MR regurgitant fractions [10]. To date, no study has evaluated whether LASr and LA stiffness derived from 3D echocardiography provide prognostic information in patients with MR.

From the prospective 3D-PRIME study, we hereby report the prevalence of altered 3D LA mechanics (peak strain in the reservoir -LASr- and contractile phase- LASct) and increased 3D LA stiffness in patients with moderate or greater primary MR and their association with occurrence of clinical events. Additionally, we explore whether measurements of 3D LA strain and stiffness provide incremental prognostic information over current guideline-recommended indicators of adverse outcomes in primary MR.

## Methods

### Study population

Data were collected in patients with significant (moderate or greater) MR and no other severe valvular heart disease enrolled prospectively in the 3D-PRIME study at the Heart Valve Clinic, Haukeland University Hospital, Bergen, Norway between 2020 and 2024. The inclusion and exclusion criteria have been previously detailed [10]. For the present analysis, we selected patients with primary MR and complete 3D-PRIME follow-up as by the predefined study protocol (2-year follow-up in patients without indication for valve intervention or follow-up to surgery/percutaneous mitral valve repair). Patients with a history of atrial fibrillation were included in the study only if atrial fibrillation had previously been documented also in the mild MR phase. Patients that had LA remodeling as lone criterium for valve intervention were not included in the analyses resulting in a final cohort size of 110 patients.

Data on symptoms, comorbidities and medical treatment were collected through patient interview at all study visits and by chart review. The heart rhythm at each study examination was ascertained based on a 12-lead electrocardiography. Moreover, we used the medical charts to identify previous episodes of atrial fibrillation. The study was conducted in accordance with the revised Declaration of Helsinki. All patients gave written informed consent, and the study was approved by the regional ethics committee (2020/106848).

### Echocardiographic measurements

The patients underwent combined 2D and 3D echocardiography using the same ultrasound equipment (Vivid E95, GE Vingmed Ultrasound, Horten, Norway), and all echocardiographic acquisitions were analyzed on Echopac workstations (version 204, GE Vingmed Ultrasound, Horten, Norway). In the small subgroup with atrial fibrillation during the examination (11 patients), only loops with acceptable rhythm regularity (variation in R-R-interval < 10%) were used in strain analyses.

### LA volumes, strain and stiffness

Details of the echocardiographic protocol have been reported [10]. In short, 3D LA remodeling was assessed using 4-to-6 beats full-volume 3D acquisitions with an average temporal resolution of 60 ± 19 vol/s. The method has previously demonstrated good intra- and interobserver agreement [11]. Additionally, the 2D LAVs and LA strain were measured in apical 4- and 2-chamber LA-focused acquisitions in line with the current recommendations [12]. By convention, the LA appendage and the ostia of the pulmonary veins were not included in the LAVs [13]. In 3D acquisitions, the automatic tracking of the LA wall was manually checked in all patients and adjusted to ensure appropriate capture of the wall motion. We report here the 3D maximum LAV (LAVmax) in absolute values as well as values indexed for body surface area (BSA). LAV was considered enlarged if 3D LAVmax/BSA ≥43ml/m^2^[5]. The LA emptying fraction was calculated from the ratio: (LAVmax-LAVmin)/LAVmax.

Peak LA strain in the reservoir (LASr) and contractile phase (LASct) was obtained semi-automatically from 2D and 3D acquisitions using dedicated software (AFI LA and 4D Auto LAQ, respectively) [14]. We classified 2D LASr as low if < 26.1% and 2D LASct as low if less negative than − 7.7%, respectively, representing the thresholds recommended by the Normal Reference Ranges for Echocardiography (NORRE) study of the European Association of Cardiovascular Imaging [15]. In the definition of low 3D LASr and 3D LASct we used the sex-specific limits of normality recently reported in healthy individuals participating in the Copenhagen City Heart Study: LASr below 18.3%/18.7% and LASct less negative than − 4.4/−3.8 in women/men [16].

A 2D and a 3D index of LA stiffness were derived from the ratio of peak transmitral early inflow velocity and the peak mitral annulus velocity during early diastole (E/e´) divided by either the 2D or the 3D LASr [17, 18]. Mitral e’ was averaged from measurements in the septal and lateral mitral annulus. The use of the mitral E/e´ in the definition of stiffness was based on the earlier reported good agreement with the mean LA pressure in the setting of both primary and secondary MR [19, 20]. 2D LA stiffness was classified as high if ≥ 0.22/0.42/0.55 in patients aged below 40, 40 to 60, and above 60 years based on data from the NORRE study [15]. We defined 3D LA stiffness as increased using a new threshold associated with higher risk of adverse outcomes in our population, as detailed below.

### Ventricular size and function

The 3D LV end-diastolic and end-systolic volumes were measured in 4-to-6 beats full-volume 3D acquisitions with a temporal resolution of 40 ± 8 vol/s [21, 22]. LV global systolic function was assessed by the Simpson´s biplane ejection fraction (2D EF) and the 2D global longitudinal strain (GLS) obtained from all three standard apical views [23]. LV GLS was considered low if less negative than − 16.7% in men and − 17.8% in women [23]. 

### MR grading

MR was graded using a multiparametric approach combining qualitative, semiquantitative and quantitative criteria. Quantification included the effective regurgitant orifice area; the regurgitant volume and the 3D regurgitant fraction (the ratio of MR regurgitant volume to the 3D LV total stroke volume) [4, 24]. In line with current recommendations, an MR regurgitant fraction above 50% was considered indicative of severe MR [4].

### Follow-up and outcomes

We examined the impact of 3D LA function on the primary outcome of the 3D-PRIME study, a composite of all-cause mortality, worsening heart failure symptoms requiring hospitalization or treatment intensification coordinated by the institutional Heart Failure Outpatient Clinic, or MR progression during follow-up reaching the threshold for mitral valve intervention (either mitral valve surgery or percutaneous repair). All deaths were ascertained from medical records. Referral to mitral intervention was based strictly on current European guidelines without taking 3D echocardiographic measures or strain indices into consideration [4]. As by the 3D-PRIME protocol, patients were followed up prospectively for 2 years or until mitral valve intervention or death.

### Statistical analyses

Statistical analyses were performed in IBM SPSS Statistics 28.0 (IBM Corp., Armonk, NY, USA) as well as R version 4.2.3 (R Foundation for Statistical Computing, Vienna, Austria). The baseline clinical characteristics as well as LA size, strain and stiffness were compared between patients who experienced the primary outcome during follow-up vs. patients without events. Findings are reported as percentages for categorical variables, mean ± standard deviation (SD) for normally distributed continuous variables, and median and interquartile range for continuous variables without a normal distribution. Comparisons between groups were performed by χ^2^ tests for categorical variables, and independent-samples t-tests or Mann-Whitney U test for continuous variables with vs. without a normal distribution.

**Fig. 1:**
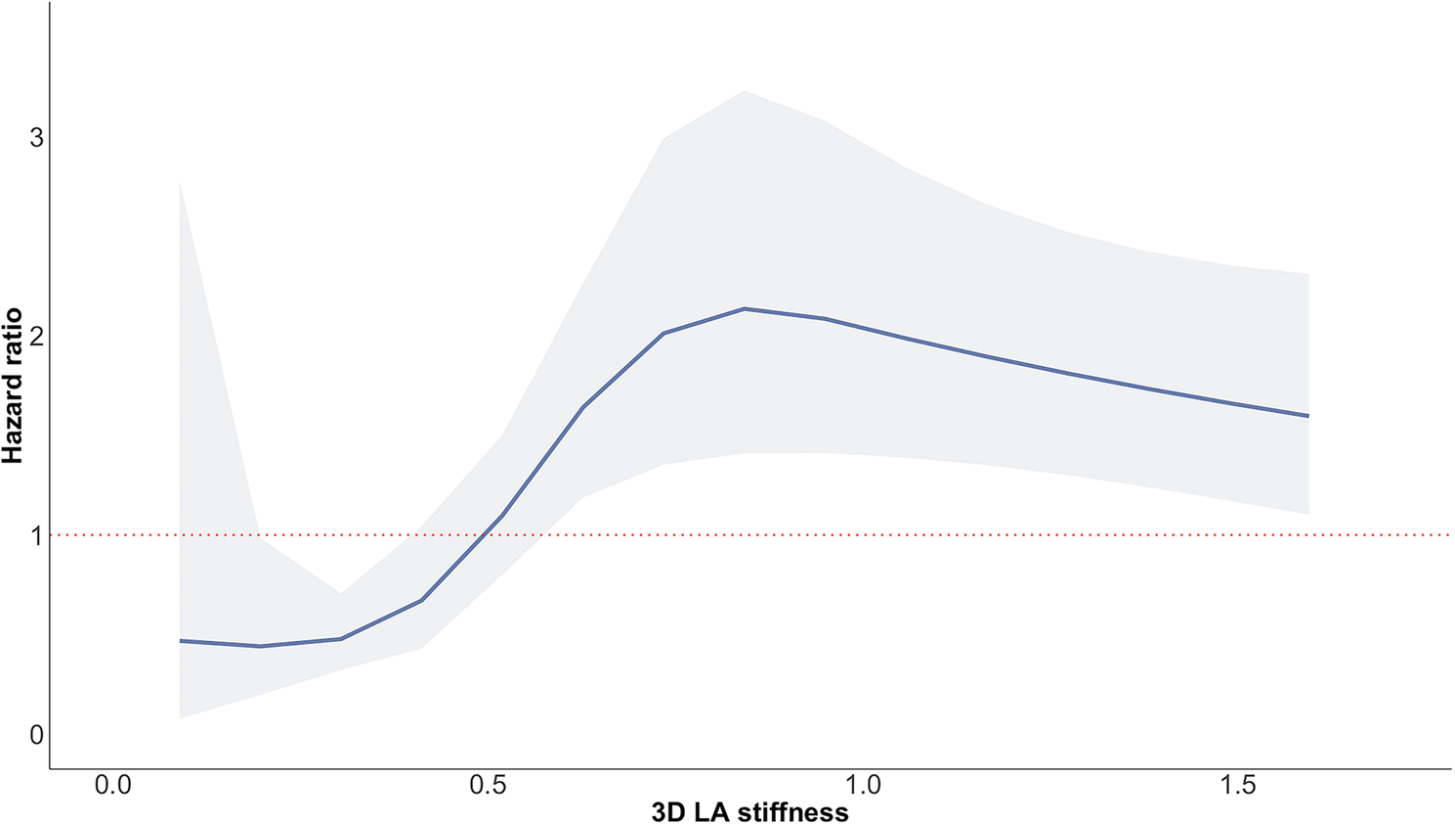
Spline curve demonstrating the hazard ratio for the occurrence of the composite outcome during follow-up according to the 3D LA stiffness. The curve shows the hazard ratio change for the occurrence of the outcome with 95% confidence intervals across a range of LA stiffness values at the baseline study echocardiogram. The threshold for increased 3D LA stiffness derived from this curve and checked using the R package Survminer was 0.50

Changes in the hazard ratio of the primary outcome across the 3D LA stiffness values were investigated by fitting a spline curve to the data (Fig. 1). The threshold of increased stiffness (as indicated by a hazard ratio above 1 in the spline curve) was also checked using the R package Survminer based upon maximal rank statistics from univariable Cox regression analysis. These tests identified 0.50 as the optimal prognostic cut-off for increased 3D LA stiffness in our population.

The impact of LA strain and stiffness on the composite outcome was evaluated in Kaplan-Meier survival analyses with log-rank test for the overall analysis, as well as in univariable and multivariable Cox regression analyses. In univariable analyses, we included clinically relevant variables and all the guideline-recommended echocardiographic criteria for MR progression that should prompt evaluation for intervention (i.e. LV EF, LV end-systolic size, LA size, the estimated systolic pulmonary artery pressure and atrial fibrillation probably due to MR progression). Only covariables associated with the primary outcome at *p* < 0.1 in univariable analyses were further included in the multivariable Cox models. Furthermore, the incremental prognostic value of 2D and 3D LASr and LA stiffness over current guidelines-recommended criteria and LV GLS (elementary model) was analysed using likelihood ratio tests and the Harrell C concordance statistic index. We assessed the change in the global χ^2^ value when each measure of LA function was added to the elementary model. Results are reported as hazard ratios with 95% confidence intervals (CI). A two-tailed *p* < 0.05 was considered significant in all analyses.

## Results

### Baseline clinical and echocardiographic characteristics

The baseline characteristics of the cohort are summarized in Table 1. The cause of primary MR was degenerative (mitral valve prolapse) in 80% of the patients. Patients that experienced events during the study (54% of the cohort) had higher heart rate, more often atrial fibrillation and used more frequently lipid-lowering medication at the baseline visit compared to patients without events. The age and sex distributions and the comorbidity profile were otherwise comparable between groups (Table 1). MR was markedly more severe among patients with events, with 73% of patients with severe MR vs. 23% of those with moderate MR at study baseline subsequently experiencing the primary outcome (*p* < 0.001). The baseline LV end-systolic volume was larger in the event group (72±23 ml vs. 64±25 ml, *p* < 0.05), while LV EF: 61% vs. 59%, and LV GLS: −18.0% vs. −17.4%, did not differ significantly between groups.

**Table 1:** Baseline clinical characteristics of the whole study population and in the groups with vs. without events during follow-up

	All (*n* = 110)	Event group (*n* = 59)	Non-event group (*n* = 51)	*p* value
Age, years	68 (56–78)	69 (57–80)	66 (54–75)	0.100
Women	34%	29%	39%	0.250
BMI, kg/m^2^	25.3 ± 3.5	25.4 ± 3.9	25.1 ± 2.9	0.625
Heart rate, bpm	67 (60–76)	71 (62–79)	63 (59–70)	**0.004**
Systolic BP, mmHg	136 (124–153)	140 (128–155)	134 (124–153)	0.242
Diastolic BP, mmHg	81 (75–88)	83 (74–92)	79 (75–83)	0.056
Atrial fibrillation at examination	10%	15%	4%	**0.048**
History of hypertension	40%	42%	37%	0.585
History of diabetes mellitus	2%	2%	2%	0.917
Coronary artery disease	17%	20%	14%	0.360
Chronic kidney disease	7%	7%	8%	0.830
Current medication:				
- Antihypertensive	57%	64%	49%	0.104
- Lipid-lowering	37%	46%	28%	**0.048**
- Antidiabetic	4%	2%	6%	0.242

*BMI* body mass index, *BP *blood pressure

Data are presented as median (25th − 75th percentiles), mean ± standard deviation, or as percentages

The *p* values in the last column are based on by χ^2^ tests for categorical variables, and independent-samples t-tests or Mann-Whitney U test for continuous variables with vs. without a normal distribution

*P* values below 0.05 are indicated in bold text

Low LASr was present in 59%/66% of the patients, and low LASct in 25%/20% when assessed by 3D vs. 2D echocardiography respectively. 2D LA stiffness was increased in 38% and 3D stiffness in 55% of the cohort. In patients subsequently experiencing events, LA was more adversely remodeled at baseline, being characterized by higher volumes, lower LA emptying fraction and more impaired LASr and LASct (Table 2). Increased 3D LA stiffness was particularly prevalent in these patients: 73% of the event group vs. 35% of the group with no events (*p* < 0.001).

**Table 2:** Baseline echocardiographic characteristics of the whole study population and in the groups with vs. without events during follow-up

	All (*n* = 110)	Event group (*n* = 59)	Non-event group (*n* = 51)		*p* value
3D LAVmax, ml	91 (72–120)	110 (87–140)	78 (64–93)	**< 0.001**	
3D LAVmax/BSA, ml/m^2^	46 (39–64)	58 (45–73)	40 (36–46)	**< 0.001**	
3D LA emptying fraction, %	42 ± 12	39 ± 11	46 ± 12	**0.003**	
2D LA emptying fraction, %	44 ± 12	41 ± 10	48 ± 13	**0.007**	
3D LASr, %	16.4 (10.8–22.8)	14.0 (10.0–19.6.0.6)	19.4 (11.2–25.0)	**0.005**	
2D LASr, %	22.1 (16.5–28.5)	22.0 (16.0–26.0)	25.2 (18.2–30.5)	**0.049**	
3D LASct, %	−7.0 (−10.1 - −3.0)	−6.0 (−7.5 - −1.8)	−8.7 (−12.0 - −5.5)	**0.001**	
2D LASct, %	−10.6 (−14.2 - −7.3)	−8.8 (−13.1 - −5.9)	−11.9 (−15.8 - −8.6)	**0.002**	
3D LA stiffness	0.58 (0.34–0.99)	0.72 (0.48–1.22)	0.39 (0.28–0.75)	**0.001**	
2D LA stiffness	0.41 (0.28–0.66)	0.51 (0.36–0.72)	0.33 (0.23–0.54)	**0.001**	
Systolic pulmonary artery pressure, mmHg	35 ± 10	35 ± 11	34 ± 10	0.834	
MR regurgitant volume (ml)	53 (31–74)	69 (55–89)	33 (22–51)	**< 0.001**	
MR effective regurgitant orifice area (mm^2^)	30 (20–47)	43 (29–61)	22 (13–30)	**< 0.001**	
MR 3D regurgitant fraction (%)	54 (36–70)	61 (51–74)	37 (26–61)	**< 0.001**	

*BSA* body surface area, *LA *left atrial, *LAV *left atrial volume. Data are presented as median (25th − 75th percentiles), mean ± standard deviation, or as percentages

The *p* values in the last column are based on independent-samples t-tests or Mann-Whitney U test for continuous variables with vs. without a normal distribution

*P* values below 0.05 are indicated in bold text

### LA strain in relation to clinical outcomes

 During a median follow-up of 24 [17–26] months, the study cohort experienced the following events: 41 mitral valve surgeries; 15 percutaneous mitral valve interventions; 8 cases of worsening heart failure; and 6 deaths. The indication for valve intervention was onset of MR-related symptoms in 79% of cases (of these, dyspnea/chest pain in 83%/17%), and significant LV remodeling with (1 patient) or without pulmonary hypertension in 21% of cases. Overall, the incidence of events was comparable between sexes (*p* = 0.25).

In Cox analysis, a dilated LA by 3D echocardiography was related to higher risk of adverse events even after 2D LAVmax/BSA was forced into the model: adjusted HR 4.0 (95% CI 1.9–8.6), *p* < 0.001.

**Fig. 2:**
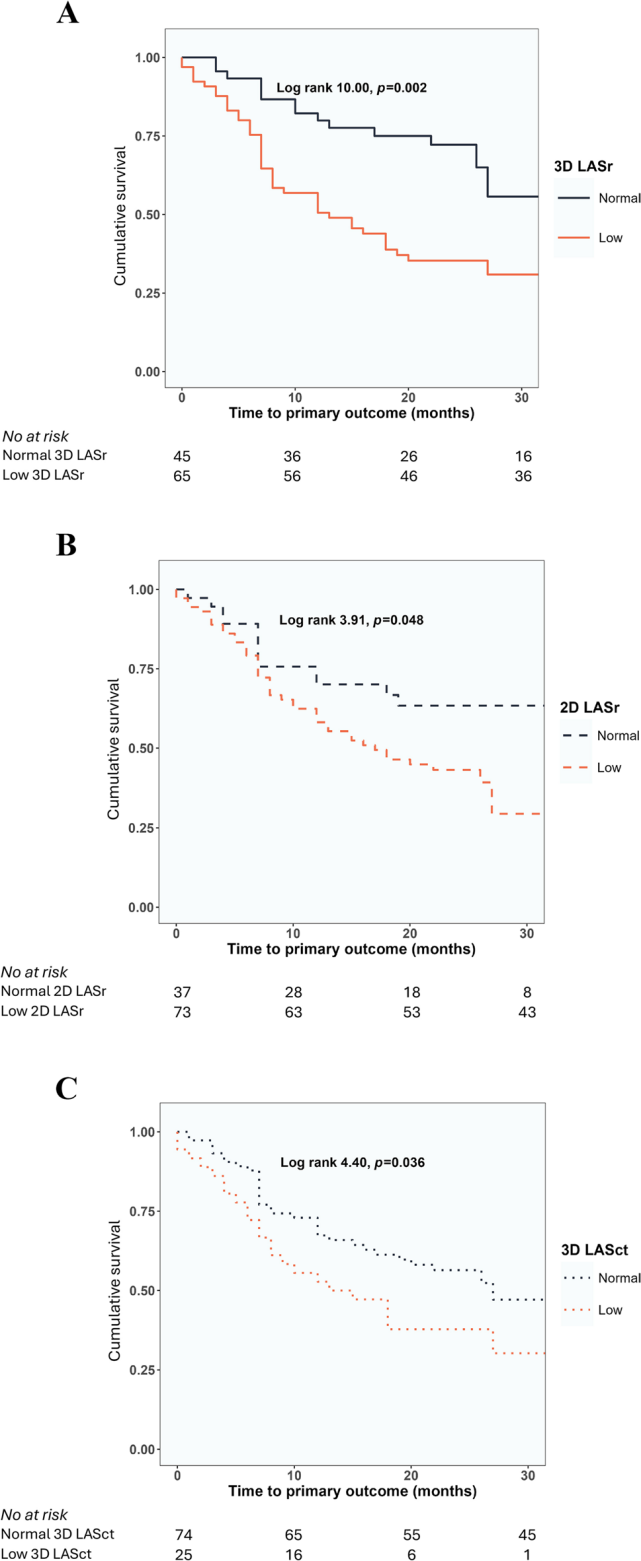
Kaplan-Meier curves for time to the composite outcome in patients with: **A**) normal vs. low 3D LASr; **B**) normal vs. low 2D LASr; **C**) normal vs. low 3D LASct. In each panel, the value of the log rank test is presented for the overall analysis with the respective *p* value

Among LA strain measures, both low 3D LASr, low 2D LASr as well as low 3D and 2D LASct predicted the primary outcome in Kaplan-Meier analyses (Fig. 2).

In univariable Cox regression analyses, both low 3D LASr and low 3D LASct were associated with increased risk of adverse events (*p* < 0.05, Table 3). In multivariable analyses, after exclusion of variables with non-significant univariable associations (*p* > 0.1) and after adjustment for LV GLS and the guideline criteria for assessment of MR progression, low 3D LASr independently predicted a 2.1-fold higher risk of adverse outcome (Table 3). In a subsequent model, use of LV end-systolic diameter instead of LV end-systolic volume did not change the association between low 3D LASr and occurrence of events. Moreover, when the analysis was restricted to patients in sinus rhythm during the examination (99 cases), low 3D LASr was still associated with a 2.2-fold (95% CI 1.1–4.6) higher risk of adverse clinical events.

**Table 3:** Predictors of the primary outcome in patients with moderate and severe primary MR. In the multivariable model, either low 3D LASr (model 1), low 3D LASct (model 2) or increased LA stiffness (model 3) in combination with clinical variables were used

Variable	Univariable analyses		Multivariable analyses					
			Model 1		Model 2		Model 3	
	HR (95% CI)	*P*	HR (95% CI)	*P*	HR (95% CI)	*P*	HR (95% CI)	*P*
Low 3D LASr	2.41 (1.35–4.30)	**0.003**	2.13 (1.05–4.31)	**0.036**	-	-	-	-
Low 3D LASct	1.71 (1.02–2.87)	**0.043**			1.50 (0.85–2.65)	0.160	-	-
Increased 3D LA stiffness	2.87 (1.58–5.21)	**0.001**					3.49 (1.64–7.39)	**0.001**
Age, years	1.01 (0.99–1.03)	0.166						
Female sex	1.19 (0.68–2.09)	0.550						
Heart rate, bpm	1.01 (0.99–1.03)	0.132						
Lipid-lowering medication	1.49 (0.89–2.49)	0.130						
AF at examination	1.35 (0.66–2.78)	0.412						
MR regurgitant fraction, %	4.20 (2.42–7.30)	**< 0.001**	1.82 (0.97–3.43)	0.063	2.22 (1.20–4.13)	**0.011**	1.74 (0.90–3.36)	0.098
LV GLS, %	0.91 (0.84–0.99)	**0.022**	0.90 (0.82–0.98)	**0.020**	0.92 (0.84–1.00.84.00)	0.055	0.85 (0.77–0.95)	**0.003**
LV EF, %	1.02 (0.99–1.04)	0.129						
LV end-systolic volume, ml	1.59 (1.12–2.27)	**0.010**	1.01 (0.99–1.02)	0.290	1.01 (0.99–1.02)	0.168	1.01 (0.99–1.02)	0.225
Systolic pulmonary artery pressure, mmHg	1.01 (0.99–1.03)	0.161						
LAVmax, ml	1.02 (1.01–1.03)	**< 0.001**	1.02 (1.01–1.02)	**< 0.001**	1.02 (1.01–1.02)	**< 0.001**	1.01 (1.00–1.02.00.02)	**0.004**

*AF* atrial fibrillation, *LA *left atrial, *LASr *left atrial reservoir strain, *LAVmax *maximum left atrial volume, *LV GLS *left ventricular global longitudinal strain, *MR *mitral regurgitation

*P *values below 0.05 are indicated in bold text

When tested in a similar multivariable Cox model, low 3D LASct did not retain its prognostic significance (Table 3).

Lower 2D LASr was also a predictor of the primary outcome after adjustment for the same variables, but with a weaker association than the one found for 3D LASr: HR 1.1 (95% CI 1.0–1.1.0.1) per 1% LASr decrease (*p* = 0.03). When both low 2D LASr and low 3D LASr were forced into the same multivariable Cox model, only 3D LASr remained significantly associated with the outcome with an adjusted HR of 2.2 (95% CI 1.1–4.5).

### LA stiffness in relation to clinical outcomes

**Fig. 3:**
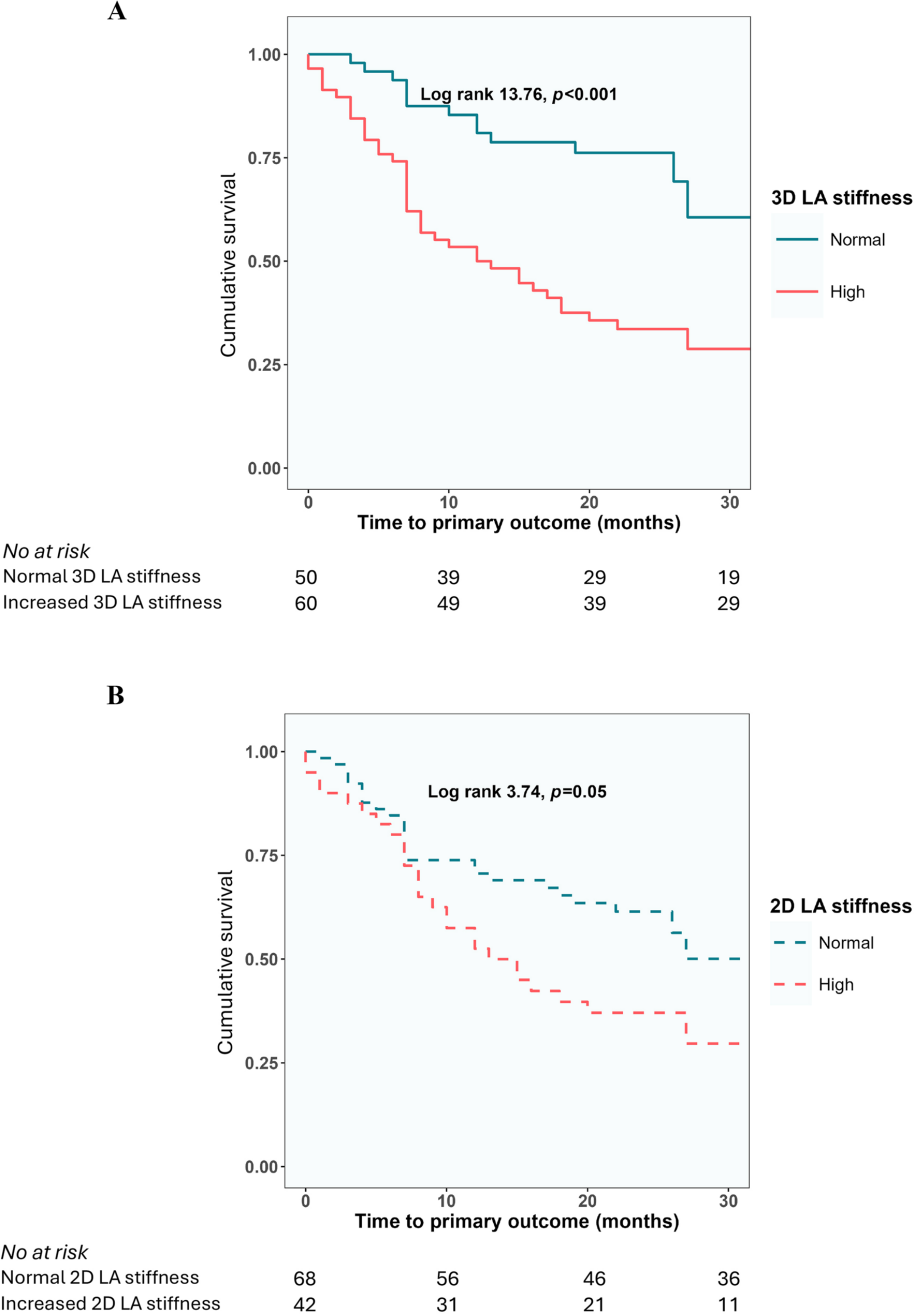
Kaplan-Meier curves for time to the composite outcome in patients with: **A**) normal vs. increased 3D LA stiffness; **B**) normal vs. increased 2D LA stiffness. In each panel, the value of the log rank test is presented for the overall analysis with the respective *p* value

As for LASr, both increased 3D and 2D LA stiffness predicted the primary outcome in Kaplan-Meier tests (Fig. 3). In multivariable Cox regression analysis, after adjustment for the same covariables as in the strain models, increased 3D LA stiffness was associated with a 3.5-fold higher risk of the primary outcome (Table 3). When the same analysis was run only in patients in sinus rhythm at the examination, the results remained unchanged with high LA stiffness associated with occurrence of adverse events by an adjusted HR 3.4 (95% CI 1.6–7.3), *p* < 0.001.

**Fig. 4:**
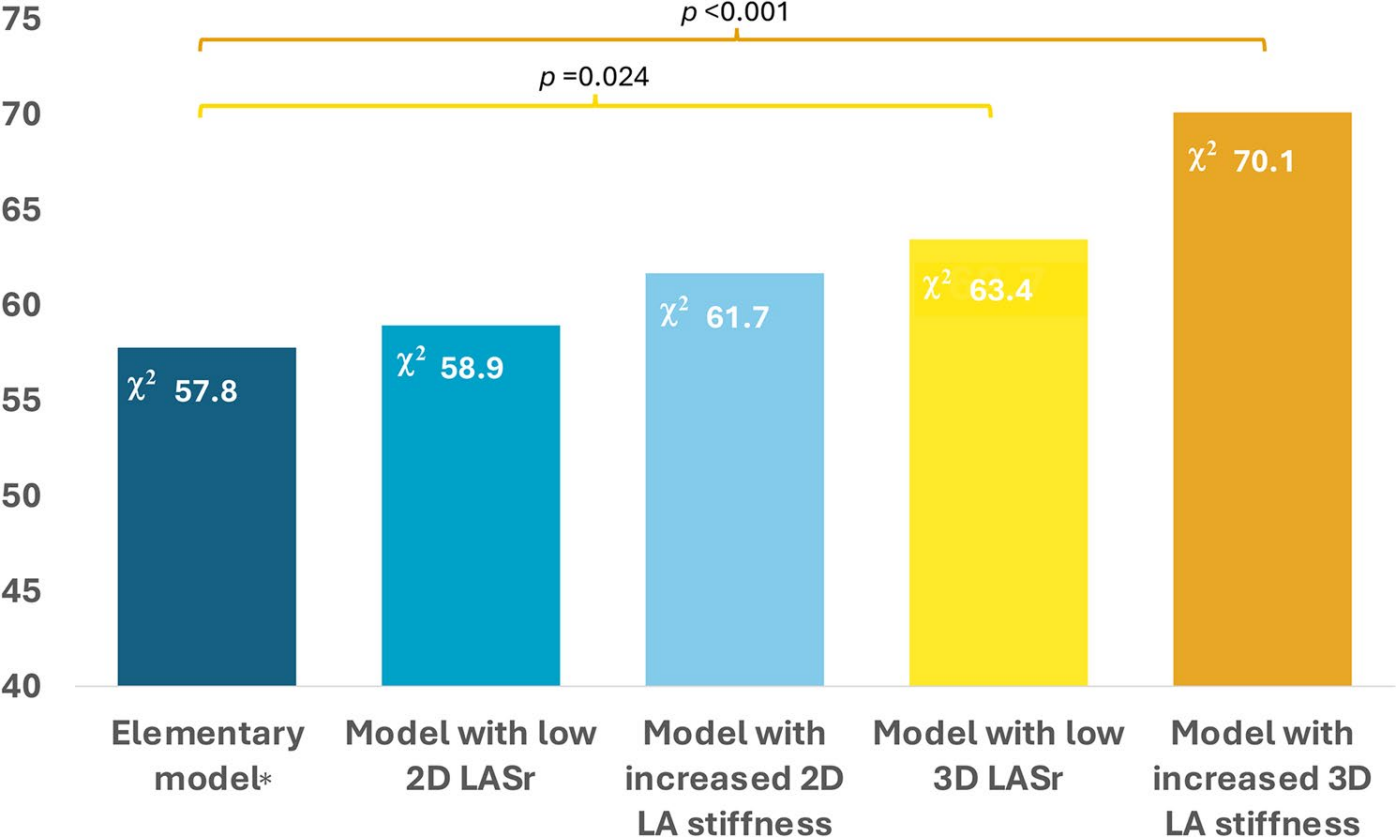
Likelihood ratio tests for the incremental prognostic value of 3D LASr and 3D LA stiffness over an *elementary clinical model (including MR severity, LV GLS, 2D LAVmax, LV EF, LV end-systolic volume, AF and estimated systolic pulmonary artery pressure). Adding either low 3D LASr or increased 3D LA stiffness to the elementary model was associated with a significant increase in the global χ^2^ value. Low 2D LASr or increased 2D LA stiffness did not have significant incremental prognostic value over the elementary model

Compared to an elementary Cox model including LV GLS and guideline criteria for intervention in primary MR, adding either low 3D LASr or increased 3D LA stiffness to the model significantly increased its predictive power (Fig. 4). On contrary, low 2D LASr or increased 2D LA stiffness did not demonstrate significant incremental value (Fig. 4). This was confirmed by no significant increase in the Harrell´s C-index when adding 2D LASr or 2D LA stiffness to the elementary model (from 0.822 to 0.829 and 0.840, respectively), but an improvement upon incorporating 3D LA stiffness (C-index 0.863, *p* < 0.05).

**Fig. 5:**
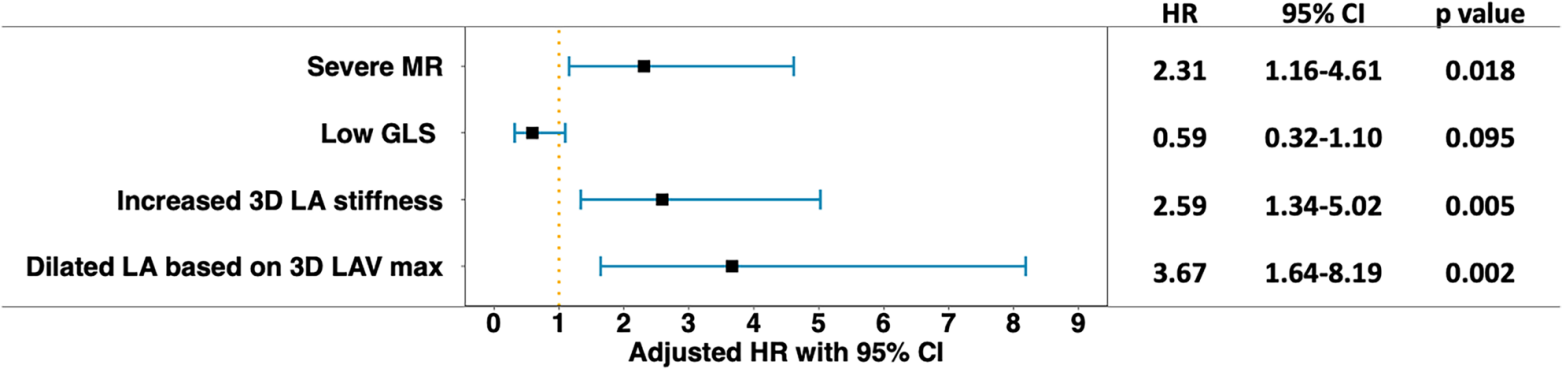
Forest plot depicting the adjusted HRs with 95% CIs for occurrence of the primary outcome for increased 3D LA stiffness, dilated LA defined from the 3D LAVmax, low LV GLS and severe MR in patients with moderate or greater primary MR

In a subsequent multivariable Cox analysis including the following predictors of outcome as categorical variables: increased 3D LA stiffness, low LV GLS, dilated LA defined from the 3D LAVmax, and severe MR, both increased LA stiffness and dilated LA were strongly associated with higher risk of adverse events (Fig. 5).

## Discussion

This study provides an in-depth analysis of 3D LA function in patients with moderate or greater primary MR in relation to the occurrence of adverse events during a 2-year prospective follow-up. Our main findings are as follows: (1) 3D indices of LA strain, particularly those reflecting the LA reservoir mechanics, predict progression of primary MR towards death, worsening heart failure, and valve intervention; (2) increased 3D LA stiffness is associated with adverse clinical outcomes in primary MR; (3) assessing 3D LASr and 3D LA stiffness, in addition to LV GLS and the current guidelines-recommended echocardiographic criteria for evaluating MR progression, may improve the prediction of adverse events in primary MR. Our findings suggest that the assessment of 3D LA remodeling in the follow-up of patients with primary MR may better reflect the impact of MR on LA structure and function compared to 2D indices and has the potential to improve risk assessment for individual patients.

### 3D LA reservoir and pump function

Enlarged LA size has long been regarded as a hallmark of chronic MR. Recently, a growing body of evidence has underscored the importance of impaired LA function as a predictor of adverse outcomes in both primary and secondary MR [6–8]. In a retrospective analysis of a large group of patients with severe primary MR undergoing mitral valve surgery, a reduced 2D LASr was found to predict increased mortality independent of the LA size highlighting the importance of combined assessment of LA size and function in MR [6]. However, in daily practice, the LA foreshortening often encountered in standard 2D apical views also influences assessment of strain with potential overestimation of 2D LASr [11]. On the other hand, LASr and LASct derived from 3D echocardiography had prior to this study not been investigated in relation to outcomes in patients with MR. Benefitting from this project’s focus on 3D acquisitions of good quality and adequate resolution, and the newly published limits of normality for 3D LA strain, we demonstrate that low 3D LASr is the strain measurement with the most robust association with clinical outcomes in primary MR. In our cohort, a low 3D LASr predicted a 2.1-fold higher rate of adverse cardiac events after adjustment for known prognosticators. Of note, the 3D LASr values were lower than the 2D ones despite the good temporal resolution of the analyzed loops. This is in line with findings in healthy subjects and underscores that thresholds of LA dysfunction are not interchangeable between 2D and 3D imaging modalities [16]. 

Patients with chronic MR that develop atrial fibrillation and thus lose the atrial pump function present with clinical deterioration and increased morbidity after mitral valve repair [25]. In hypertensive individuals, more than mild MR has been reported to be associated with impaired 2D LASct [26]. In one study of LA mechanics in patients with MR of different etiologies, subjects with primary MR had impaired pump function (assessed by the LA strain rate) compared to controls both during exercise and in the recovery period [27]. The prognostic relevance of assessing LASct, particularly 3D LASct, in patients with primary MR has until now been largely unexplored. In our cohort, 3D LASct was univariably associated with adverse events, but did not have incremental prognostic value over established prognosticators as MR regurgitant fraction, LA and LV size and LV GLS (*p* = 0.07). However, future mechanistic studies of the interplay between the LA contraction, mitral annulus function, and MR severity might shed new light on the significance of changes in LA pump function during MR progression.

Interestingly, LA size has earlier been found to be underestimated by 2D echocardiography compared to computed tomography, cardiac magnetic resonance or 3D echocardiography [5, 28]. The LA has indeed a complex shape described as either grossly cuboidal or the fusion of two truncated cones of different heights and its geometric particularities are likely not to be fully captured by 2D measurements. Enlarged 3D LAVmax was in our analyses the LA size indicator with the most robust association with adverse cardiac events, supporting the measurement of 3D volumes in patients with MR.

### 3D LA stiffness

One of the main determinants of LASr is the degree of LA stiffness. At baseline in 3D-PRIME, 2D LA stiffness was increased in one-third of patients, probably reflecting the accumulating wall fibrosis in the setting of long-lasting volume overload as previously demonstrated in an experimental-computational setting using a canine model [10, 29]. Also in a small cohort of 46 patients with severe MR, a lower LASr was strongly correlated with a higher degree of LA fibrosis [9]. A 3D index of LA stiffness, derived from 3D LASr, has to our knowledge not been previous reported in patients with primary MR. In our population, increased LA stiffness was more prevalent when assessed by 3D than by 2D echocardiography, and significantly more common in patients subsequently experiencing adverse events. Furthermore, in contrast to its 2D counterpart, increased 3D LA stiffness showed incremental predictive value beyond guideline criteria for intervention in primary MR, possibly demonstrating an increased sensitivity of 3D parameters in detecting adverse LA remodeling. In an assessment of risk based on 4 categorical indices, enlarged LA and increased 3D LA stiffness were the two echocardiographic measurements strongest associated with higher risk of adverse clinical events. Interestingly, 2D LA stiffness has been reported to be higher among patients with severe primary MR during exercise compared to controls, and highest in patients with severe secondary MR [27]. Future studies on the reversibility of LA stiffness after mitral intervention and its impact on postoperative outcomes are warranted.

### Clinical perspective

In moderate or greater primary MR, a combined assessment of LA size and function using 3D echocardiography identifies a significant group of patients with advanced LA remodeling and an increased risk of adverse events over a 2-year follow-up. Notably, evaluating 3D LA reservoir function and stiffness may enhance the prediction of death, heart failure worsening, and MR progression beyond currently used echocardiographic indicators. This suggests that 3D measures may have higher sensitivity in detecting LA myopathy. Future multicenter trials are needed to determine if low LA reservoir function or increased LA stiffness warrants earlier intervention in significant primary MR.

### Study limitations

The 3D-PRIME study is conducted at one heart valve clinic by investigators with experience within 3D echocardiography and the present findings should therefore be confirmed in larger, multicenter populations with longer follow-up periods. Of note, due to the technological progress during the last decade, 3D echocardiography of the LA has become an investigator-friendly tool possible to use in large community-based studies [16]. The threshold of increased 3D LA stiffness in healthy subjects has not yet been established, and the cut-off of 0.50 identified in our cohort needs further validation before adoption in clinical practice. Our patients were elderly with a median age of 68 years. Future studies should investigate the threshold of increased 3D LA stiffness in younger individuals. Our cohort was entirely Caucasian, and prospective studies of 3D LA remodeling in MR should also be conducted in multiethnic populations [16]. Women amounted for 34% of our study population and were less referred to mitral surgery than men. The sex-specific association between 3D LA changes in primary MR and adverse outcomes needs to be further analyzed in a larger cohort. There was a low burden of atrial fibrillation in our patients, and its presence was not associated with outcomes. Moreover, in analyses conducted only in patients in sinus rhythm, low 3D LA function and stiffness maintained their prognostic relevance. The exact cause of death could not be established in all patients; therefore, we report the all-cause mortality. However, the incidence of death was low during the 2-year study follow-up. Finally, future studies combining 3D echocardiography and other imaging modalities, such as cardiac magnetic resonance, will enable the validation of 3D LA stiffness against a different technique. They might also provide new opportunities for development of more precise methods of MR quantification.

## Conclusion

In patients with moderate or greater primary MR, impaired 3D LA function is associated with an increased risk of disease progression towards death, worsening heart failure and mitral valve intervention. Low 3D LASr and increased LA stiffness have the potential to enhance risk assessment in primary MR when combined with current guideline-recommended echocardiographic criteria for intervention. Our findings provide a foundation for future multicenter studies to further investigate the value of routine assessment of LA remodeling using 3D echocardiography in the follow-up of patients with primary MR.

## Data Availability

The data that support the findings of this study are available from the corresponding author upon reasonable request.

## Abbreviations

3D-PRIME: Three-Dimensional Echocardiography and Cardiovascular Prognosis in Mitral Regurgitation
BMI: Body mass index
BP: Blood pressure
BSA: Body surface area
CV: Cardiovascular
EF: Ejection fraction
GLS: Global longitudinal strain
LA: Left atrium or left atrial
LASr: Left atrial reservoir strain
LASct: Left atrial contractile strain
LAV(s): Left atrial volume(s)
LAVmax: Maximum left atrial volume
LAVmin: Minimum left atrial volume
LV: Left ventricle or left ventricular

## References

[1] Rusinaru D, Tribouilloy C, Grigioni F, Avierinos JF, Suri RM, Barbieri A, et al. Left atrial size is a potent predictor of mortality in mitral regurgitation due to flail leaflets: results from a large international multicenter study. Circ Cardiovasc Imaging. 2011;4:473–81. 21737598 10.1161/CIRCIMAGING.110.961011

[2] Essayagh B, Antoine C, Benfari G, Messika-Zeitoun D, Michelena H, Le Tourneau T, et al. Prognostic implications of left atrial enlargement in degenerative mitral regurgitation. J Am Coll Cardiol. 2019;74:858–70. 31416529 10.1016/j.jacc.2019.06.032

[3] Le Tourneau T, Messika-Zeitoun D, Russo A, Detaint D, Topilsky Y, Mahoney DW, et al. Impact of left atrial volume on clinical outcome in organic mitral regurgitation. J Am Coll Cardiol. 2010;56:570–8. 20688212 10.1016/j.jacc.2010.02.059

[4] Praz F, Borger MA, Lanz J, Marin-Cuartas M, Abreu A, Adamo M, Marsan NA, Barili F, Bonaros N, Cosyns B, De Paulis R, Gamra H, Jahangiri M, Jeppsson A, Klautz RJM, Mores B, Perez-David E, Poss J, Prendergast BD, Rocca B, Rossello X, Suzuki M, Thiele H, Tribouilloy CM, Wojakowski W, and Group EESD. 2025 ESC/EACTS guidelines for the management of valvular heart disease. Eur J Cardiothorac Surg. 2025;67:1–109.

[5] Badano LP, Miglioranza MH, Mihaila S, Peluso D, Xhaxho J, Marra MP, et al. Left atrial volumes and function by Three-Dimensional echocardiography: reference Values, Accuracy, Reproducibility, and comparison with Two-Dimensional echocardiographic measurements. Circ Cardiovasc Imaging. 2016;9:1–12. 10.1161/CIRCIMAGING.115.004229 27412658

[6] Stassen J, van Wijngaarden AL, Butcher SC, Palmen M, Herbots L, Bax JJ, et al. Prognostic value of left atrial reservoir function in patients with severe primary mitral regurgitation undergoing mitral valve repair. Eur Heart J Cardiovasc Imaging. 2022;24:142–51. 35301525 10.1093/ehjci/jeac058 PMC9762939

[7] Cramariuc D, Alfraidi H, Nagata Y, Levine RA, van Kampen A, Andrews C, Hung J. Atrial dysfunction in significant atrial functional mitral regurgitation: phenotypes and prognostic implications. Circ Cardiovasc Imaging. 2023;16:e015089. 37158081 10.1161/CIRCIMAGING.122.015089 PMC10187627

[8] Stassen J, Namazi F, van der Bijl P, van Wijngaarden SE, Kamperidis V, Marsan NA, Delgado V, Bax JJ. Left atrial reservoir function and outcomes in secondary mitral regurgitation. J Am Soc Echocardiogr. 2022;35:477–85. e3. 35074443 10.1016/j.echo.2022.01.007

[9] Cameli M, Lisi M, Righini FM, Massoni A, Natali BM, Focardi M, et al. Usefulness of atrial deformation analysis to predict left atrial fibrosis and endocardial thickness in patients undergoing mitral valve operations for severe mitral regurgitation secondary to mitral valve prolapse. Am J Cardiol. 2013;111:595–601. 23211360 10.1016/j.amjcard.2012.10.049

[10] Berg-Hansen CE, Sindre RB, Grymyr LMD, Rogge B, Valeur AE, Urheim S, Hung J, Cramariuc D. Sex differences in left atrial volumes, mechanics, and stiffness in primary mitral regurgitation-a combined 2D and 3D echocardiographic study. Eur Heart J Cardiovasc Imaging. 2024;25:1118–26. 38469654 10.1093/ehjci/jeae072 PMC11288747

[11] Nabeshima Y, Kitano T, Takeuchi M. Reliability of left atrial strain reference values: a 3D echocardiographic study. PLoS One. 2021;16:e0250089. 33852637 10.1371/journal.pone.0250089 PMC8046190

[12] Lang RM, Badano LP, Mor-Avi V, Afilalo J, Armstrong A, Ernande L, Flachskampf FA, Foster E, Goldstein SA, Kuznetsova T, Lancellotti P, Muraru D, Picard MH, Rietzschel ER, Rudski L, Spencer KT, Tsang W, Voigt JU. Recommendations for cardiac chamber quantification by echocardiography in adults: an update from the American society of echocardiography and the European association of cardiovascular imaging. Eur Heart J Cardiovasc Imaging. 2015;16:233–70. 25712077 10.1093/ehjci/jev014

[13] Thomas L, Muraru D, Popescu BA, Sitges M, Rosca M, Pedrizzetti G, Henein MY, Donal E, Badano LP. Evaluation of left atrial size and function: relevance for clinical practice. J Am Soc Echocardiogr. 2020;33:934–52. 32762920 10.1016/j.echo.2020.03.021

[14] Badano LP, Kolias TJ, Muraru D, Abraham TP, Aurigemma G, Edvardsen T, D’Hooge J, Donal E, Fraser AG, Marwick T, Mertens L, Popescu BA, Sengupta PP, Lancellotti P, Thomas JD, Voigt JU. Industry r and reviewers: this document was reviewed by members of the ESDC. Standardization of left atrial, right ventricular, and right atrial deformation imaging using two-dimensional speckle tracking echocardiography: a consensus document of the EACVI/ASE/Industry task force to standardize deformation imaging. Eur Heart J Cardiovasc Imaging. 2018;19:591–600. 29596561 10.1093/ehjci/jey042

[15] Sugimoto T, Robinet S, Dulgheru R, Bernard A, Ilardi F, Contu L, et al. Echocardiographic reference ranges for normal left atrial function parameters: results from the EACVI NORRE study. Eur Heart J Cardiovasc Imaging. 2018;19:630–8. 29529180 10.1093/ehjci/jey018

[16] Yafasov M, Olsen FJ, Skaarup KG, Lassen MCH, Johansen ND, Lindgren FL, Jensen GB, Schnohr P, Mogelvang R, Sogaard P, Biering-Sorensen T. Normal values for left atrial strain, volume, and function derived from 3D echocardiography: the Copenhagen City heart study. Eur Heart J Cardiovasc Imaging. 2024;25:602–12. 38261728 10.1093/ehjci/jeae018

[17] Kurt M, Wang J, Torre-Amione G, Nagueh SF. Left atrial function in diastolic heart failure. Circ Cardiovasc Imaging. 2009;2:10–5. 19808559 10.1161/CIRCIMAGING.108.813071

[18] Sindre RB, Gerdts E, Putaala J, Grymyr LMD, Midtbo H, Almeida AG, et al. Association of left atrial stiffness with risk of cryptogenic ischemic stroke in young adults. JACC Adv. 2024;3:100903. 38939654 10.1016/j.jacadv.2024.100903 PMC11198254

[19] Ritzema JL, Richards AM, Crozier IG, Frampton CF, Melton IC, Doughty RN, Stewart JT, Eigler N, Whiting J, Abraham WT, Troughton RW. Serial doppler echocardiography and tissue doppler imaging in the detection of elevated directly measured left atrial pressure in ambulant subjects with chronic heart failure. JACC Cardiovasc Imaging. 2011;4:927–34. 21920328 10.1016/j.jcmg.2011.07.004

[20] Agricola E, Galderisi M, Oppizzi M, Melisurgo G, Airoldi F, Margonato A. Doppler tissue imaging: a reliable method for estimation of left ventricular filling pressure in patients with mitral regurgitation. Am Heart J. 2005;150:610–5. 16169349 10.1016/j.ahj.2004.10.046

[21] Muraru D, Badano LP, Peluso D, Dal Bianco L, Casablanca S, Kocabay G, Zoppellaro G, Iliceto S. Comprehensive analysis of left ventricular geometry and function by three-dimensional echocardiography in healthy adults. J Am Soc Echocardiogr. 2013;26:618–28. 23611056 10.1016/j.echo.2013.03.014

[22] Muraru D, Spadotto V, Cecchetto A, Romeo G, Aruta P, Ermacora D, Jenei C, Cucchini U, Iliceto S, Badano LP. New speckle-tracking algorithm for right ventricular volume analysis from three-dimensional echocardiographic data sets: validation with cardiac magnetic resonance and comparison with the previous analysis tool. Eur Heart J Cardiovasc Imaging. 2016;17:1279–89. 26647080 10.1093/ehjci/jev309

[23] Sugimoto T, Dulgheru R, Bernard A, Ilardi F, Contu L, Addetia K, et al. Echocardiographic reference ranges for normal left ventricular 2D strain: results from the EACVI NORRE study. Eur Heart J Cardiovasc Imaging. 2017;18:833–40. 28637227 10.1093/ehjci/jex140

[24] Zoghbi WA, Adams D, Bonow RO, Enriquez-Sarano M, Foster E, Grayburn PA, et al. Recommendations for noninvasive evaluation of native valvular regurgitation: a report from the American society of echocardiography developed in collaboration with the society for cardiovascular magnetic resonance. J Am Soc Echocardiogr. 2017;30:303–71. 28314623 10.1016/j.echo.2017.01.007

[25] Ngaage DL, Schaff HV, Mullany CJ, Barnes S, Dearani JA, Daly RC, Orszulak TA, Sundt TM. 3rd. Influence of preoperative atrial fibrillation on late results of mitral repair: is concomitant ablation justified? Ann Thorac Surg. 2007;84:434–42. 10.1016/j.athoracsur.2007.04.036 17643612

[26] Tang SS, Shi R, Yang ZG, Wang J, Min CY, Yan WF, Zhang Y, Li Y. Incremental effect of mitral regurgitation on left atrial dysfunction and atrioventricular interaction in hypertensive patients by MRI. J Magn Reson Imaging. 2023;58:1125–36. 36733221 10.1002/jmri.28604

[27] Sugimoto T, Bandera F, Generati G, Alfonzetti E, Barletta M, Losito M, Labate V, Rovida M, Caracciolo M, Pappone C, Ciconte G, Guazzi M. Left atrial dynamics during exercise in mitral regurgitation of primary and secondary origin: pathophysiological insights by exercise echocardiography combined with gas exchange analysis. JACC Cardiovasc Imaging. 2020;13:25–40. 30878440 10.1016/j.jcmg.2018.12.031

[28] Mor-Avi V, Yodwut C, Jenkins C, Kuhl H, Nesser HJ, Marwick TH, Franke A, Weinert L, Niel J, Steringer-Mascherbauer R, Freed BH, Sugeng L, Lang RM. Real-time 3D echocardiographic quantification of left atrial volume: multicenter study for validation with CMR. JACC Cardiovasc Imaging. 2012;5:769–77. 22897989 10.1016/j.jcmg.2012.05.011

[29] Bouwmeester S, van Loon T, Ploeg M, Mast TP, Verzaal NJ, van Middendorp LB, et al. Left atrial remodeling in mitral regurgitation: a combined experimental-computational study. PLoS One. 2022;17:e0271588. 35839240 10.1371/journal.pone.0271588 PMC9286246

